# Obesity as a Risk Factor for the Severity of COVID-19 in Pediatric Patients: Possible Mechanisms—A Narrative Review

**DOI:** 10.3390/children11101203

**Published:** 2024-09-30

**Authors:** Dana Elena Mîndru, Elena Țarcă, Heidrun Adumitrăchioaiei, Dana Teodora Anton-Păduraru, Violeta Ștreangă, Otilia Elena Frăsinariu, Alexandra Sidoreac, Cristina Stoica, Valentin Bernic, Alina-Costina Luca

**Affiliations:** 1 Department of Mother and Child Medicine, University of Medicine and Pharmacy “Gr.T.Popa”, 700115 Iasi, Romania; mindru.dana@umfiasi.ro (D.E.M.); dana.anton@umfiasi.ro (D.T.A.-P.); violeta.streanga@umfiasi.ro (V.Ș.); frasinariu.otilia@umfiasi.ro (O.E.F.); alina.luca@umfiasi.ro (A.-C.L.); 2 Department of Surgery II—Pediatric Surgery, University of Medicine and Pharmacy “Gr.T.Popa”, 700115 Iasi, Romania; 3 Department of Pediatrics, University of Medicine, Pharmacy, Sciences and Technology “George Emil Palade”, Târgu Mureș, Str. Gheorghe Marinescu Nr. 38, 540136 Târgu Mureș, Romania; heidrun.adumitrachioaiei@umfst.ro; 4 Emergency Clinical Hospital for Children “Sfanta Maria” Iasi, 700309 Iași, Romania; sidoreac.alexandra@email.umfiasi.ro (A.S.); stoica.cristina@email.umfiasi.ro (C.S.); 5 Department of Surgery II, “Saint Spiridon” Hospital, University Street, No 16, 700115 Iasi, Romania; bernic.valentin@email.umfiasi.ro

**Keywords:** obesity, inflammation, viral infections, multisystem involvement, severity

## Abstract

Obesity, the current pandemic, is associated with alarming rises among children and adolescents, and the forecasts for the near future are worrying. The present paper aims to draw attention to the short-term effects of the excess adipose tissue in the presence of a viral infection, which can be life-threatening for pediatric patients, given that the course of viral infections is often severe, if not critical. The COVID-19 pandemic has been the basis of these statements, which opened the door to the study of the repercussions of obesity in the presence of a viral infection. Since 2003, with the discovery of SARS-CoV-1, interest in the study of coronaviruses has steadily increased, with a peak during the pandemic. Thus, obesity has been identified as an independent risk factor for COVID-19 infection and is correlated with a heightened risk of severe outcomes in pediatric patients. We sought to determine the main mechanisms through which obesity is responsible for the unfavorable evolution in the presence of a viral infection, with emphasis on the disease caused by SARS-CoV-2, in the hope that future studies will further elucidate this aspect, enabling prompt and effective intervention in obese patients with viral infections, whose clinical progression is likely to be favorable.

## 1. Introduction

Obesity is a significant global health issue with severe repercussions on the physical and mental quality of life [1,2]. Unfortunately, for a long time, obesity has been considered exclusively as an adult disease, leading to the neglect of its impact on the pediatric population. Consequently, nowadays, childhood and adolescent obesity have become major public health concerns, associated with alarming prevalence rates and worrying forecasts for the coming years [3,4].

We define obesity as the excess of adipose tissue in the body, therefore drawing attention to the importance of psychological repercussions [5]. According to studies, a relationship has been identified between obesity and depression, with obese individuals suffering twice as much from depression versus normal-weight people [6]. The disruption of the hypothalamic–pituitary–adrenal (HPA) axis that occurs in response to stress is a common feature in both obesity and depression. Additionally, stigmatization is an important risk factor to which obese people are subjected, which entails the onset of multiple pathologies in the psychiatric sphere, including panic attacks, anxiety, behavioral disorders, and varying degrees of depression [7,8,9,10]. 

In the long term, obesity is widely known to be a risk factor for numerous chronic, non-communicable pathologies, such as metabolic syndrome, diabetes mellitus, cardiovascular disease, atherosclerosis, and stroke. Importantly, achieving a normal weight status has been shown to mitigate these risks [5,11,12,13]. 

The short-term effects of obesity will be detailed by apparatus and systems, in regard to the viral infection determined by COVID-19.

Lifestyle changes in recent decades, with reduced time spent outdoors in the form of exercise and increased time spent using technology, along with poor-quality nutrition, rich in junk food, and highly processed and inadequate intakes of essential macro- and micronutrients, are the primary contributors to the rising widespread occurrence of overweightness and obesity [14,15,16]. The COVID-19 pandemic only increased this percentage by entrenching these unbalanced and unhealthy lifestyle patterns [3]. 

In Wuhan, the first case of COVID-19 infection was initially declared to be a case of pneumonia. Subsequently, the World Health Organization defined COVID-19 as a disease in February 2020, followed by the onset of the COVID-19 pandemic on 11 March 2020. This development, entirely unexpected by both the general population and the medical community, opened new horizons for research [17,18]. Therefore, it was not long before the first studies began to draw attention to the fact that patients who were either overweight or obese had a worse outcome than those with a normal weight [19]. Although the large number and severity of COVID-19 cases in adults led to a research focus on overweight adult patients, it became evident that despite the generally milder evolution of the disease in the pediatric group, there were numerous cases of severe infection. These severe cases, often associated with overweight children, quickly became a significant area of research [20].

We thus aim to highlight the effects of SARS-CoV-2 on different apparatus and systems among overweight or obese patients, with the expectation of raising awareness about the negative impact of obesity, not only as a pathology in its own right but also as an acute risk factor in the presence of viral infections, which have occupied an important place in the diagnostic scene worldwide in the last decade [21,22]. Also, we intend to explore the role of lifestyle changes during the pandemic, especially poor nutrition and inactivity, in increasing the risk of obesity and its effect on COVID-19 severity. Another topic we want to target is the relationship between obesity and mental health issues and their influence in viral infections. In this narrative review, we used existing studies from PubMed and Web of Science to exemplify the mechanism of action of obesity in COVID-19 and the implication in other associated diseases. 

At the same time, we seek to contribute to the global fight against pediatric obesity because today’s obese child, and especially the obese adolescent, is tomorrow’s obese adult, further increasing the prevalence of non-communicable diseases. These diseases prematurely remove individuals from the labor market and impose substantial long-term costs on healthcare systems, due to complicated treatments and frequent hospitalizations [2,7,23].

## 2. The Evolution and Genetic Structure of Coronaviruses

Coronaviruses are a large family of viruses under the order Nidovirales, family Coronaviridae, and subfamily Orthocoronavirinae. They possess a single-stranded RNA structure, having the longest continuous genomes among all RNA viruses. The SARS-CoV-2 genome is 26 to 32 kb in length, encoding 16 non-structural proteins, 4 structural proteins, and 11 accessory proteins [24,25,26,27]. Affecting both mammals and birds, interest in the study of coronaviruses gained momentum following the discovery of SARS-CoV-1 in 2003. The identification of MERS-CoV in Saudi Arabia in 2012 further fueled research on this family of viruses [28]. The seventh identified coronavirus, SARS-CoV-2, led to the emergence of a global pandemic.

Theories regarding the transmission of this virus from bats to humans are divided; some suggest the involvement of intermediate hosts, while others suggest direct transmission [29,30,31]. Approximately one year after its initial identification, SARS-CoV-2 began to be detected in various variants and sub-variants, which have raised significant concerns among health specialists [32].

## 3. Respiratory Distress in Obese Patients with COVID-19 Infection

In obese patients, including children and adolescents, normal respiratory function is often compromised. Due to the fact that the lungs are one of the main targets of the SARS-CoV-2 virus and that obesity poses higher risks for infected patients, this should always be kept in mind. Obesity impairs blood oxygenation and becomes even more critical when SARS-CoV-2-induced damage reduces alveolar exchange zones. Excess abdominal adiposity exerts pressure on the lungs through the diaphragm, restricting breathing movements and impairing ventilation at the lung bases, potentially leading to decreased blood oxygen saturation [33,34]. In addition, obese patients exhibit reduced lung volume and respiratory muscle strength, as well as decreased forced expiratory volume and forced vital capacity [35,36]. Vital capacity and total lung capacity may also be lessened in patients with severe obesity [37,38]. Certain obesity-related comorbidities, such as obstructive sleep apnea and the high prevalence of asthma among obese children, may contribute to an increased risk of lung infections. With regard to asthma, the inflammatory mechanisms linked to leptin and interleukin-2 (IL-2)—which contribute to the high incidence and severity of the disease in children—are also implicated in the severity of COVID-19 infection. Finally, together with the lung function impairment, children who suffer from obesity have poor exercise endurance, perpetuating the vicious circle [39].

## 4. Cardiovascular Involvement in Obese Patients with COVID-19 Infection

Even very young children exhibit anatomical changes in the heart associated with obesity, such as left ventricular hypertrophy, which correlates with the obesity grade and blood pressure rates, in addition to other structural changes [40]. Blood pressure is elevated in children and adolescents, increasing the risk of endothelial damage, which is considered one of the fundamental processes in the pathophysiology of COVID-19 infection [40,41]. Children, and especially obese children, who are treated for hypertension with specific drugs that either inhibit the activity of the angiotensin-converting enzyme (ACE2) or act as blockers on the angiotensin receptors, manifest an elevated expression of ACE2, thereby enhancing susceptibility to SARS-CoV-2 infection [42]. This particular endothelial modification can be found in any degree of obesity, from mild to severe. Narrowing of the internal layer of the arteries—the intima—is observed in children suffering from obesity, indicating the possibility of premature atherosclerosis. 

Arterial stiffening is associated with reduced nitrogen function and chronic oxidative stress, which are involved in the changes associated with severe forms of COVID-19, such as venous thromboembolism, endothelial inflammation, myocarditis, severe acute respiratory syndrome (SARS), and multiple organ failure syndrome (MOFS) [43]. Data from the latest post-mortem surveys reveal the presence of coronavirus components in endothelial cells, potentially via the viral utilization of ACE2 at the endothelial level. In these cases, inflammatory cell accumulation, stagnation of venous blood in the pulmonary venules, and inflammation of the endothelial lining in the intestinal blood vessels have been observed [43,44]. Leptin—found in great amounts in obese individuals—impairs the endothelium in a way that decreases the production of nitric oxide and increases the expression of monocyte chemotactic protein-1, thus facilitating the inflammatory infiltration in vascular cells. In addition, perivascular adipose tissue plays a role in vasoconstriction, as well as in endothelial dysfunction by producing inflammatory mediators, by reducing nitric oxide generation and by promoting oxidative stress [43].

An additional significant risk that obese people, including children and adolescents with COVID-19 infection, may encounter is the development of a coagulopathy with an unfavorable prognosis. In particular, the chronic inflammation associated with this condition inhibits essential anticoagulant factors, for instance, tissue factor pathway inhibitor, antithrombin, and protein C, while stimulating the production of procoagulant factors, such as adhesion molecules (P-selectin) and tissue factor. 

Moreover, the inflammatory process is linked to elevated thrombin production and platelet activation, consequently elevating the likelihood of thrombotic processes. In grave SARS-CoV-2 infections, venous thromboembolism is a major risk, as many patients present with elevated D-dimer levels, and others feature clinical symptoms suggestive of disseminated intravascular coagulation (DIC) [45]. Furthermore, in COVID-19-infected patients, virus-induced endothelial cell dysfunction may lead to hypercoagulability through excessive thrombin generation and the inhibition of fibrinolysis. In severe forms of the disease, hypoxia may also increase the risk of thrombosis by increasing blood viscosity through a hypoxia-induced transcription factor-dependent signaling pathway. This was confirmed by performing post-mortem dissection and lung analysis, which demonstrated obstruction and micro-thrombi in the pulmonary vessels of small caliber of a patient with COVID-19 [46,47]. 

## 5. Obesity, Metabolic Impairment, and COVID-19 Infection

**Figure 1:**
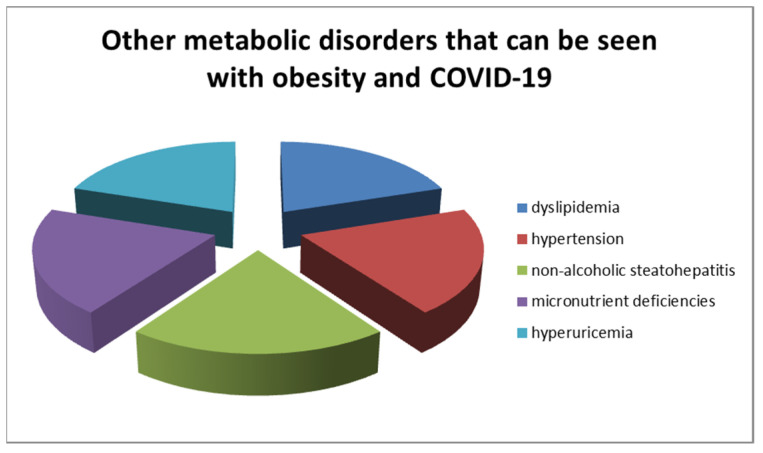
Metabolic disorders that can be seen with obesity and COVID-19.

The connection between obesity and elevated insulin levels leads to an increased insulin resistance in peripheral tissues. This condition may be accompanied by other metabolic disorders, such as hypertension, dyslipidemia, non-alcoholic steatohepatitis, hyperuricemia, micronutrient deficiencies, and increased oxidative stress (Figure 1). During an intense immune response, such as that triggered by COVID-19 infection, pancreatic beta cells are further engaged in the production of large quantities of insulin, which can be challenging, given that these cells are already functioning at full capacity [48]. The dynamics between SARS-CoV-2 and ACE2 may also contribute to pancreatic beta-cell destruction. Even if blood glucose remains within normal limits, obesity may be associated with several metabolic disorders [49,50].

## 6. Renal Impairment in Obese Patients with COVID-19 Infection 

Obesity can also target the kidneys, inducing several structural, metabolic, and hemodynamic changes, leading to an overall alteration of their functional reserve. An increase in the kidneys’ weight and volume constitutes another concern, the main cause being the ectopic deposition of adipose tissue in the renal sinus. Lipid deposition induces oxidative stress, which further favors inflammation, endothelial dysfunction, cell hypertrophy, apoptosis, increased mesangial matrix, and renal fibrosis [51,52].

Renal physiological responses to obesity include high renal plasma flow and glomerular filtration rate, elevated filtration fraction, and increased water and sodium reabsorption from the proximal tubules. These changes induce glomerular stress, as well as tubular and glomerular hypertrophy, which subsequently lead to subnephrotic-grade proteinuria and secondary glomerular sclerosis, ultimately developing chronic kidney disease [53]. As aforementioned, obesity relates to increased insulin production and insulin resistance, conditions that stimulate mesangial expansion and activate the renin–angiotensin–aldosterone system. This activation, mediated by the vasoconstrictive effect of angiotensin II on the renal arterioles, promotes endothelin-1 synthesis, further exacerbating mesangial membrane expansion, sodium retention, and vasoconstriction of the renal arterioles [54,55]. Tumor necrosis factor plays a pivotal role in the development of renal fibrosis, while the intracellular lipid level exerts toxic effects on the kidney cells, compromising mitochondrial structure and function, thereby contributing to the evolution of glomerulosclerosis and the progression of renal disease [52]. Additionally, increased body weight and, consequently, reduced urinary pH lead to the heightened excretions of oxalate, sodium, phosphate, and uric acid. This condition sets the stage for the formation of urinary lithiasis. The development of certain renal neoplasms has also been linked to obesity [56]. Furthermore, SARS-CoV-2 has been identified as causing acute kidney injury in up to 25% of patients with severe infection, particularly in those with pre-existing medical issues, positioning it as a standalone risk factor for mortality [56].

Pathophysiologically, COVID-19-associated acute kidney injury could be related to COVID-specific mechanisms. These include direct viral cytotoxicity via ACE2, highly expressed in renal tissues, which facilitates the internalization of SARS-CoV-2, and dysregulation of the renin–angiotensin–aldosterone system (RAAS), an inflammatory response characterized by elevated pro-inflammatory cytokines and thrombotic events [55]. The pathogenesis is further complicated by thrombotic events and acute tubular necrosis secondary to endothelial dysfunction [57,58].

## 7. Obesity, Mental Health, and COVID-19 Infection

**Figure 2:**
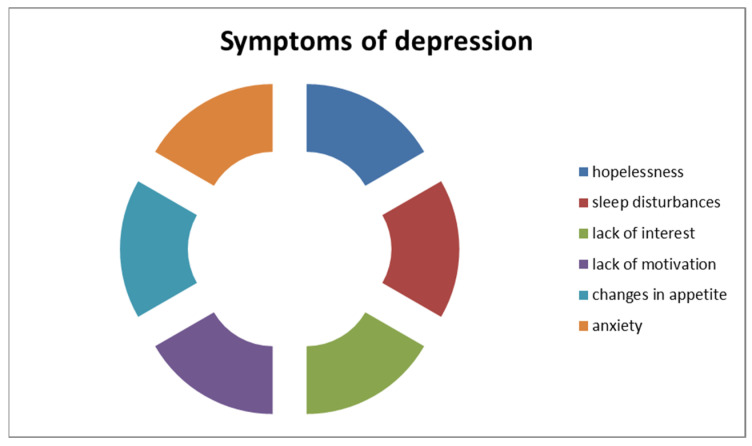
Symptoms of depression.

**Figure 3:**
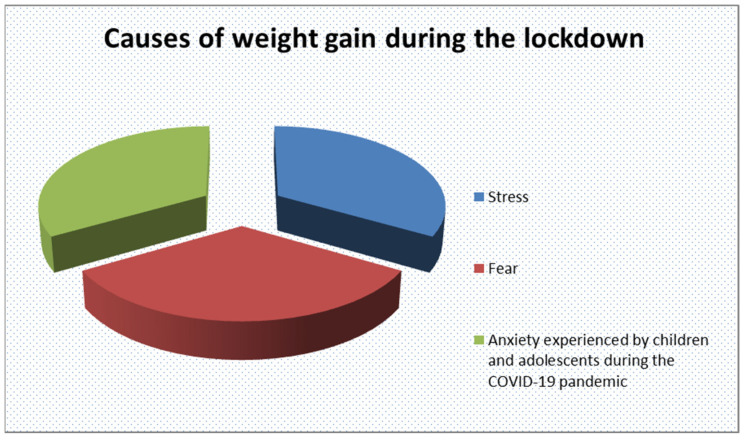
Causes of weight gain during the lockdown.

Mental health concerns, such as depression and anxiety, are also potentially significant comorbidities of obesity [10]. Pediatricians should specifically inquire about symptoms of depression, including hopelessness, sleep disturbances, lack of interest or motivation, and changes in appetite, as well as anxiety, which may be exacerbated by loneliness, isolation, and uncertainty [59,60,61] (Figure 2). The COVID-19 pandemic came with high levels of stress, fear, and anxiety registered among children and adolescents, with its lockdown aggravating this mental status (Figure 3). As prior studies have demonstrated the complex relationship between mental health and obesity, it is safe to say that most likely, the pandemic-induced emotional imbalance contributed to a degree of weight gain in the pediatric population [62,63].

In the first place, stress is responsible for stimulating chronic cortisol secretion, a major culprit in weight gain. In the second place, an extended period of stress can contribute to depression, which, combined with home isolation, lack of physical activity, and poor eating habits, generally exacerbates weight gain and obesity. This, consequently, results in social stigma, stress, and further seclusion [11,61,62,63,64]. Stress and anxiety in children and adolescents, both personally experienced and parent-reported, include limited knowledge about the mechanisms of SARS-CoV-2 spread; possible serious consequences of the infection; the unavailability of effective treatment; social seclusion; significant shifts in family dynamics, leading to worsened dysfunctional elements; financial strain resulting from job loss or unemployment; and difficulties in using the essential technology for academic activities and homework tasks [4,8,65].

With its imposed restrictions aimed at controlling the high rates of virus transmissibility, the COVID-19 pandemic has had a major impact on society, affecting people’s lives, regardless of age [3,23]. These restrictions led to increased social isolation, a sedentary lifestyle, and the development of eating disorders [57]. Moreover, the inadequate production of cortisol and pro-inflammatory cytokines has been associated with delays in children’s mental and cognitive development, obesity, asthma, recurrent infections, diabetes mellitus, and even premature death [66,67,68].

The ICD-10 defines nonspecific anxiety as a category of psychiatric disorders, characterized by pervasive feelings of anxiety, often accompanied by fear and physical symptoms associated with anxiety [69].

Many children rely on school-provided meals that are nutritionally balanced to meet their macronutrient needs. However, during the COVID-19 pandemic, school closures and the transition to online learning disrupted this access, and meals were provided by the family or purchased by the children themselves [70,71,72]. The combination of inactivity, social isolation, and heightened anxiety during this period contributed to weight gain in children and adolescents.

## 8. Discussion

The implications of viral infections on an overweight organism have been extensively studied, with well-established findings that the immune response, as well as the organism’s response to the influenza vaccine, is compromised in such cases [73]. 

Research by Zhang et al. during the H1N1 influenza pandemic associated leptin resistance with a more severe course of the flu. Leptin, a cytokine produced in adipose tissue, is less active, despite being present in higher quantities in obese people [74]. This association between leptin resistance and the severity of a viral infection may be attributed to leptin’s supportive role in the maturation, development, and function of B cells, as well as its roles in modulating lymphocytes and inhibiting the CD8+ T cell response [74]. Obesity is recognized as an independent risk factor for SARS-CoV-2 infection, and it is also identified as a predictor associated with a positive laboratory test, according to a study conducted in a pediatric population in Mexico [75,76]. 

A cohort study found that general and/or abdominal obesity is associated with a threefold increased risk of developing COVID-19, compared to individuals of normal weight. It is important to note that in this study, no statistically significant relationship was found between gender and the association of obesity with the risk of COVID-19 infection [77]. Chronic inflammation, such as that caused by obesity, further elevates the risk of developing severe infections. For instance, a study conducted in New York involving 50 pediatric patients with COVID-19 identified obesity as the most significant risk factor for requiring respiratory assistance [78].

A survey conducted in Israel from 16 February 2020 to 20 December 2021, encompassing 444,868 records, underscores the heightened risk of SARS-CoV-2 infection among overweight children and recommends prioritizing them in the vaccination process to prevent severe infection [79]. 

Additionally, a retrospective analysis of 319 children with SARS-CoV-2 infection and severe acute respiratory syndrome found that obesity (*p* < 0.001) and diabetes mellitus were significantly more prevalent in patients requiring hospitalization [80]. The correlation of obesity with severe or even critical COVID-19 infection was further corroborated by a study of 264 children hospitalized in a Canadian medical facility [19]. 

A meta-analysis undertaken in 2023 by Sheri Madigan et al. analyzed data from 40.807 children and adolescents in the pre-COVID-19 period and 33,682 in the post-COVID-19 period. The results indicated that symptoms associated with depression were more prevalent in the female population and in children and adolescents from middle- and high-income families [64]. This finding suggests a higher susceptibility attributed to the female gender to developing eating disorders associated with anxiety and depressive states. Regarding age, adolescents were more affected by stress and depression, compared to younger children, likely due to their greater awareness and understanding of the existing situation [62]. 

At the same time, adolescents have access to various information sources and are more liable to form their own opinions, unlike younger patients, who are typically protected by their parents and family. A 2021 meta-analysis by Lu Ma et al. identified problems such as depression, anxiety, sleep-related pathologies, and post-traumatic stress disorder (PTSD) as being more commonly observed in female adolescents, compared to their male counterparts and younger children [70]. These mental health disorders, including depression, insomnia, anxiety, and PTSD, have been linked to weight gain and eating disorders, with eating often serving as a compensatory mechanism during times of stress.

In the United States, from March to July 2020, over 30 million children benefited from free school meals, resulting in an increase from 32.6% to 36% in food insecurity and nutrition gaps [23,81].

The prevalence of COVID-19 infection was higher among overweight and obese children in low- and middle-income areas, underscoring the importance of focusing not only on the quantity but also the quality of nutritional management. Unfortunately, children in regions with low to middle incomes are at a greater risk of developing obesity and multiple other pathologies, exacerbated by poor socioeconomic conditions [63,82].

### 8.1. Limitations

We encountered several limitations in our study. There was a limited number of clinical trials that study the relationship between obesity and viral infection severity in pediatric populations, so it restricted the generalizability of our conclusions. The data of many studies targeting children were extrapolated from the adult studies. Additionally, the evolving nature of the virus during the COVID-19 pandemic could affect outcomes because of the different strains [43]. Another limitation was the presence of multiple methods of measuring obesity, which created inconsistencies when comparing results across studies [52]. Moreover, comorbidities like metabolic disorders and asthma were not consistently accounted for in influencing the severity of COVID-19 infections [78].

### 8.2. Prospects for Future Research

It would be beneficial to explore more long-term consequences of COVID-19 infections in obese pediatric patients because of their elevated risks of developing severe comorbidities later in life [25]. Another subject that should be considered in future research is investigating the interaction between cytokines and immune system cells in pediatric patients with obesity. This could provide deeper insights into why these patients experience worse outcomes [43]. Finally, future research in preventing and managing obesity in children by improving lifestyle changes is necessary to mitigate the risk of severe outcomes [19].

## 9. Conclusions

Obesity, by sustaining a state of low-grade inflammation, creates a favorable environment for the development of severe viral infections. Studies and analyses of the mechanisms of action related to obesity in association with COVID-19 disease suggest that obesity and viral infections, particularly COVID-19, share common metabolic and inflammatory response pathways. The obesity pandemic persists, even after the COVID-19 pandemic has waned, underscoring the dire necessity for interventions to address this global health crisis. Preventing obesity should begin at the preconception stage, emphasizing the value of proper nutrition and lifestyle of the future mother.

We believe that future large studies are essential to more comprehensively investigate the relationship between obesity and viral infections in the pediatric population.

## Data Availability

Data sharing not applicable.
